# Supplementary material for: Kindlin-1 regulates IL-6 secretion and modulates the immune environment in breast cancer models
Source: eLife. 2023 Mar 8;12:e85739. doi: 10.7554/eLife.85739 (PMC10023156; doi:10.7554/eLife.85739)
Supplement: Figure 1—figure supplement 1—source data 1. [file elife-85739-fig1-figsupp1-data1.pdf]

Unedited raw gel for Figure 1-Figure supplement 1A

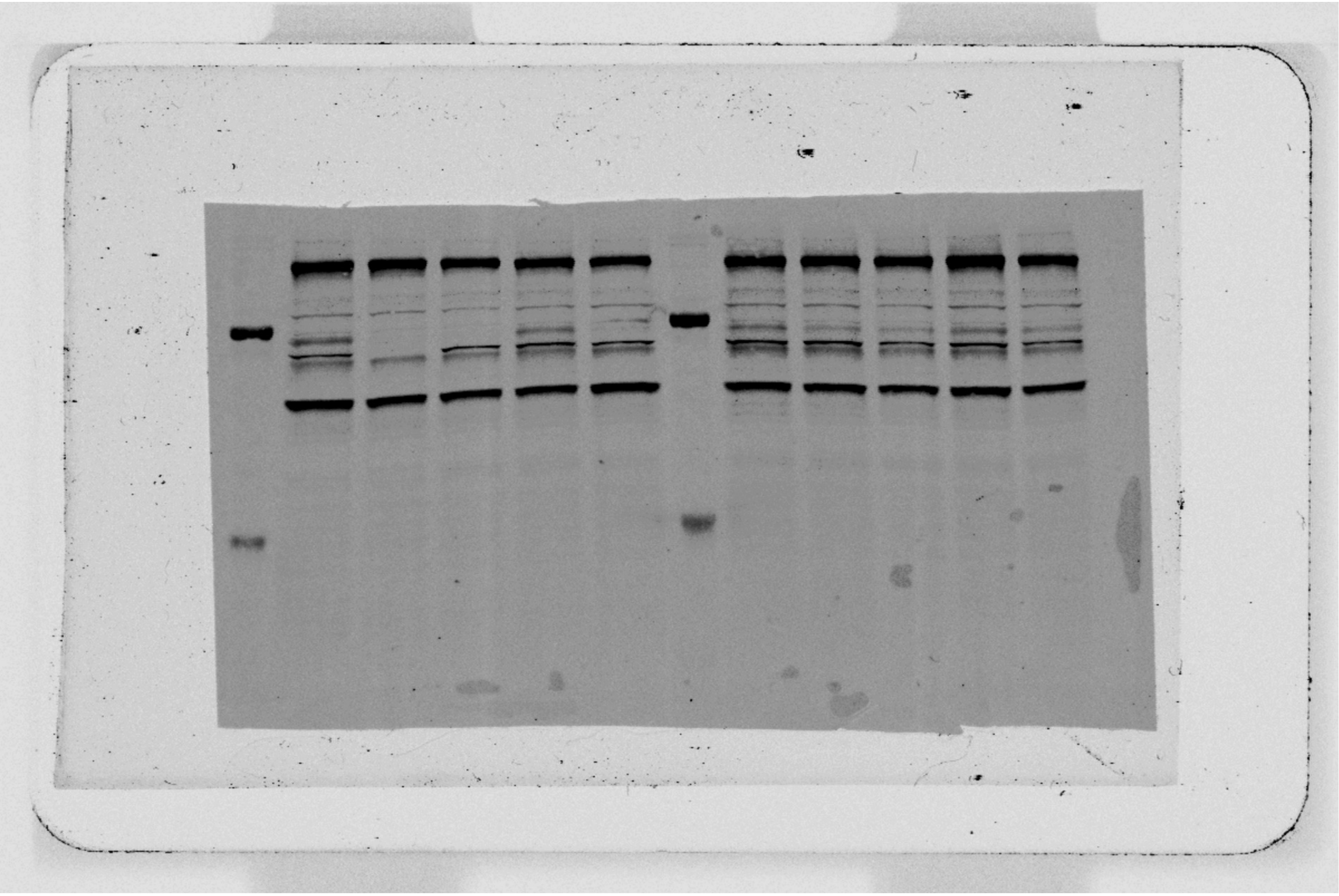

Labelled gel for Figure 1-Figure supplement 1A

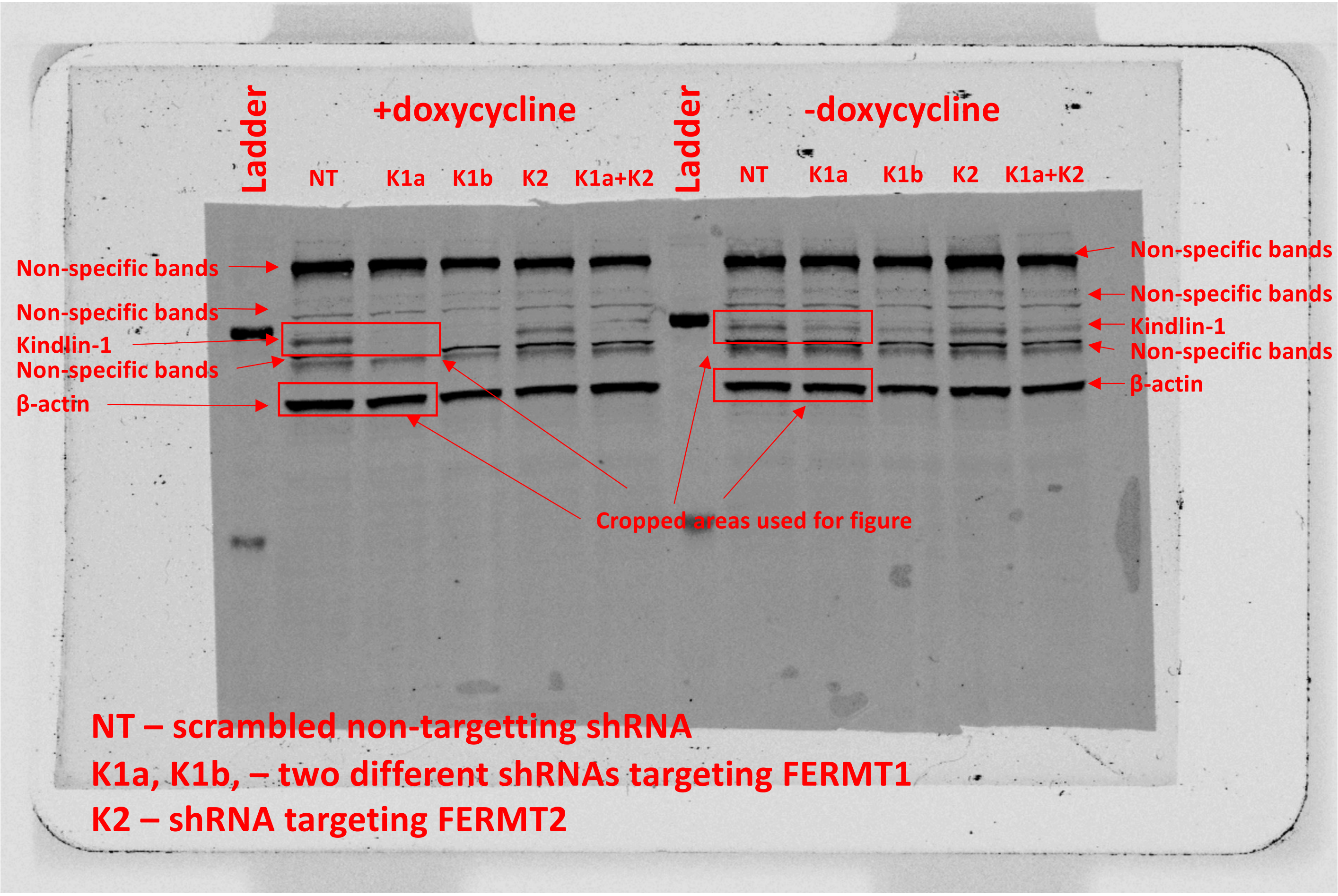

Unedited raw gel for Figure 1-Figure supplement 1C

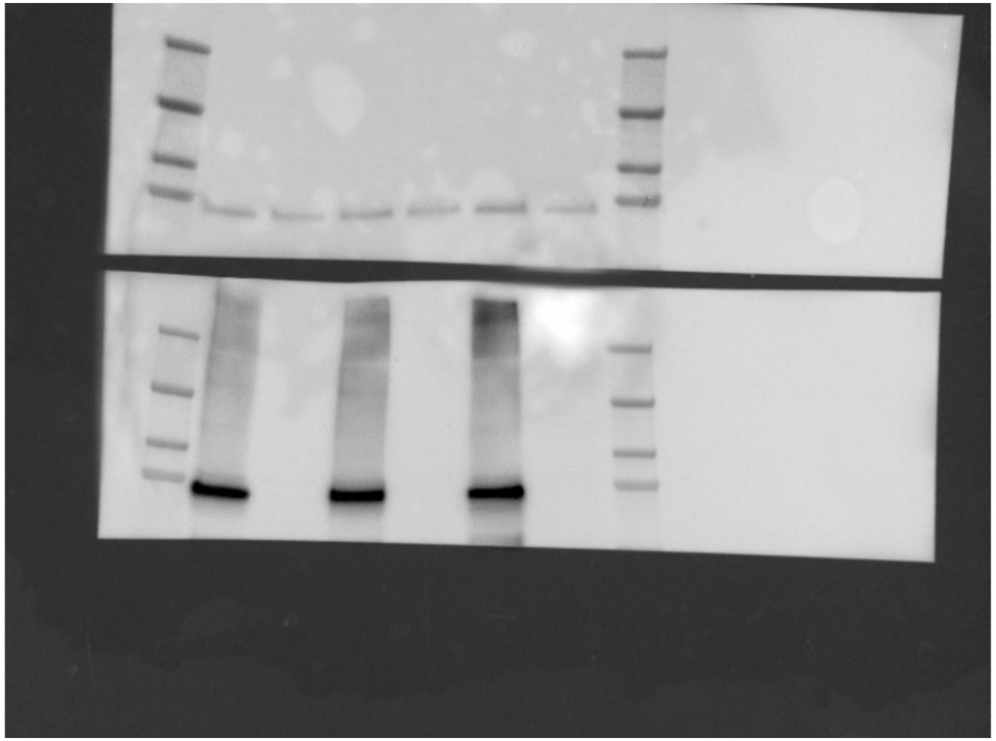

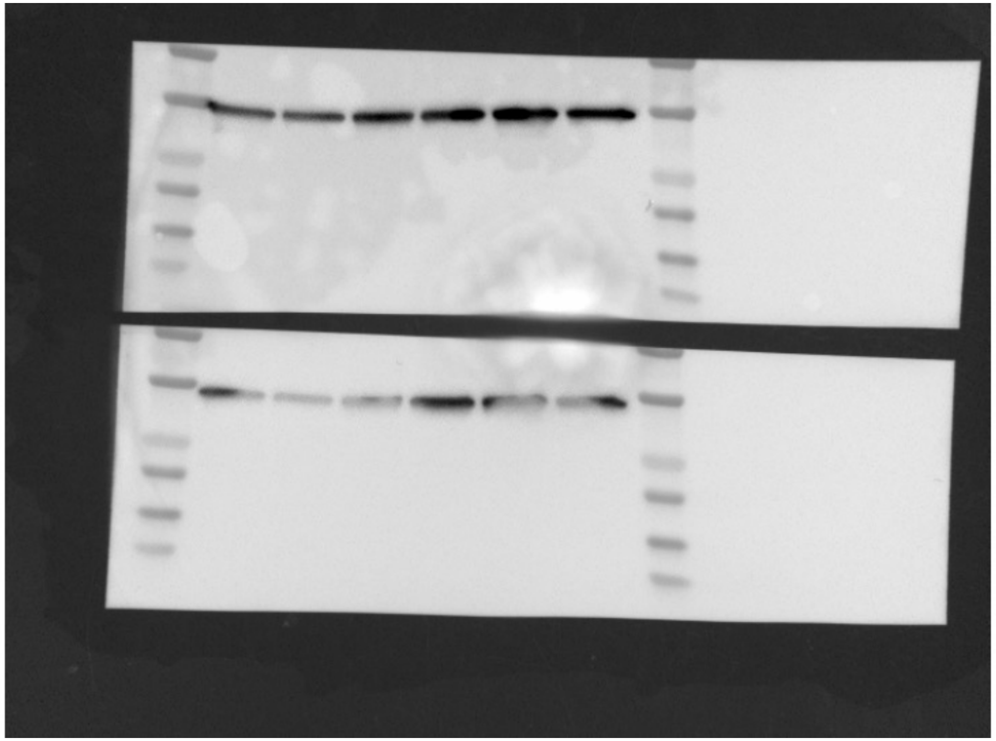

Unedited raw gel for Figure 1-Figure supplement 1C

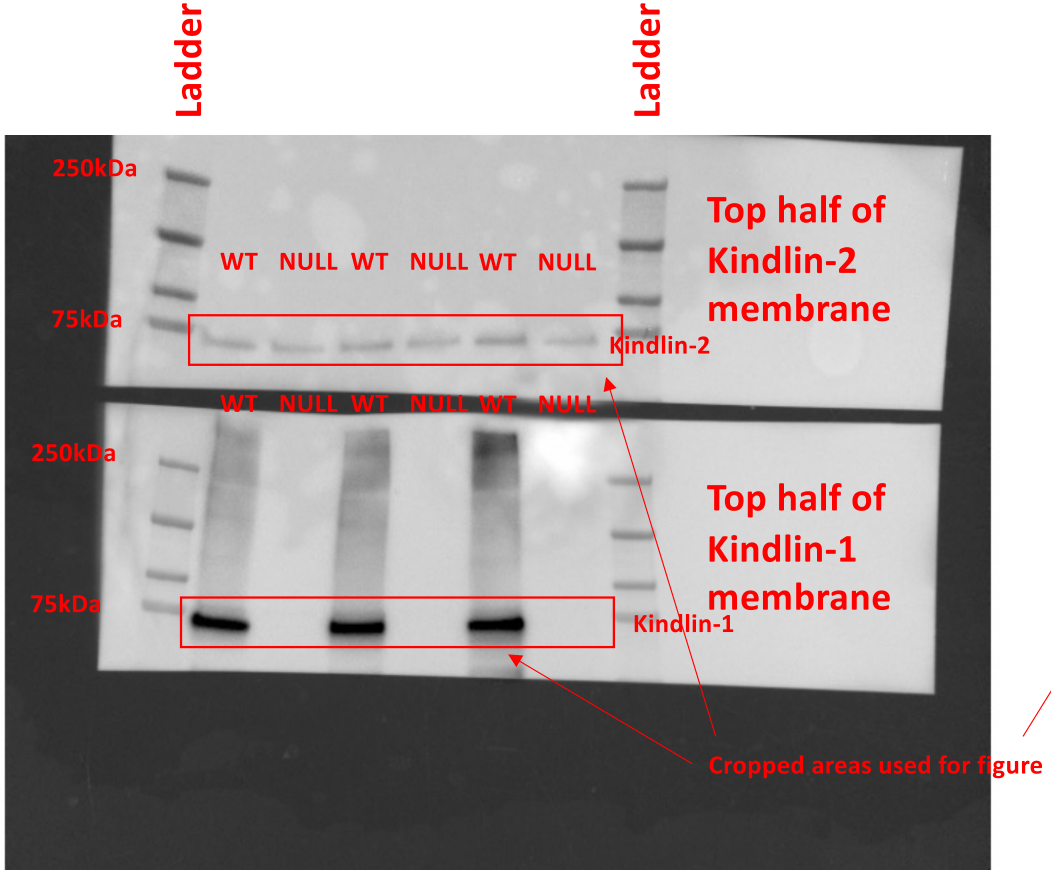

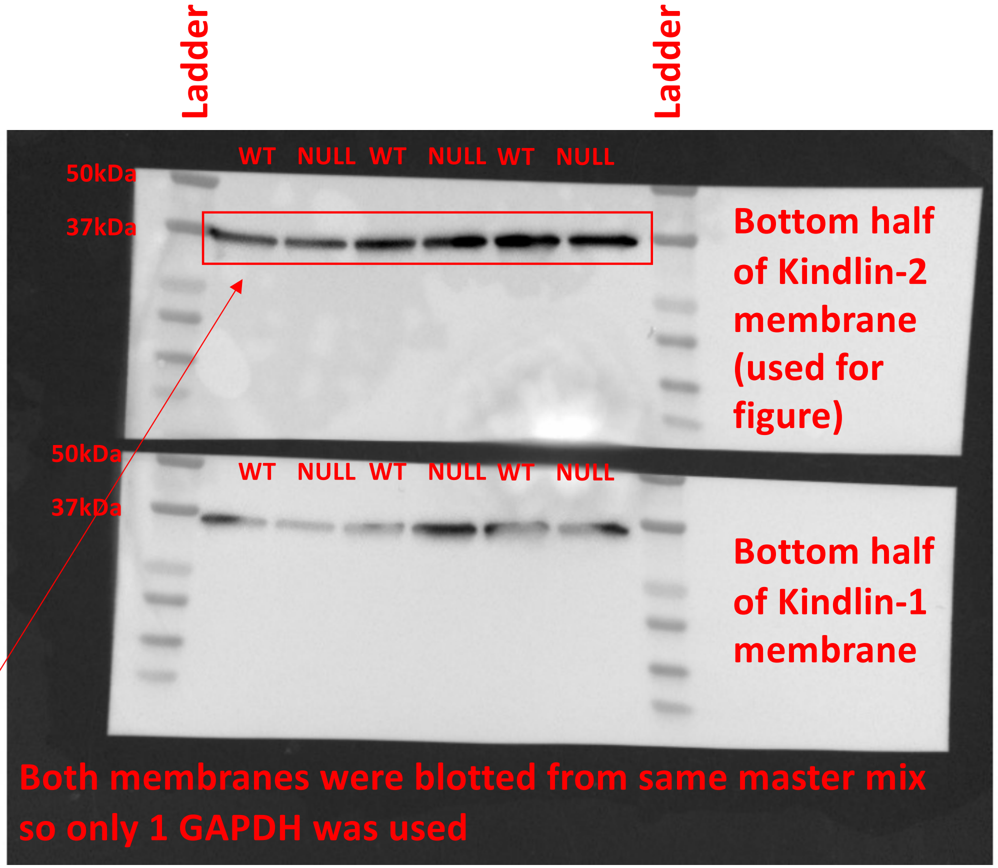

Both are merges with colorimetric images to show ladder. For figure, just the band image was used
